# Epidemiological Study of Oral Health among Children and Adolescent Schoolchildren in Melilla (Spain)

**DOI:** 10.3390/healthcare11142086

**Published:** 2023-07-21

**Authors:** Marta Hernandez-Donadeu, David Ribas-Pérez, Diego Rodriguez Menacho, Paloma Villalva Hernandez-Franch, Ignacio Barbero Navarro, Antonio Castaño-Séiquer

**Affiliations:** 1 Private Practice, 52001 Melilla, Spain; 2 Department of Stomatology, University of Seville, 41013 Seville, Spainacastano@us.es (A.C.-S.); 3 Servicio Andaluz de Salud, 41004 Andalucia, Spain

**Keywords:** oral health, Melilla, oral health survey, inequities in health, schoolchildren

## Abstract

Dental epidemiological studies are essential for analysing and evaluating the population’s health state and dental treatments provided, as well as for planning future oral health programme activities and interventions based on their findings. In order to determine the health condition of children and adolescents in connection to the prevalence of caries, caries indices (decayed and filled teeth (dft) for primary teeth and decayed, missing and filled teeth (DMFT) in permanent teeth) and periodontal indices (community periodontal index (CPI)), oral exams of children and adolescents aged 6, 12 and 15 from selected schools were conducted. To assess the achievement of the oral health objectives set for Spain in 2020, these data were compared with those acquired at the national level. At 6 years of age, 278 children were examined, the prevalence of caries was 55.6%, the dft index was 2.77 (±3.44), the DMFT was 0.19 (±0.16), the restorative index (RI) was 4.62%, and the significant caries (SiC) index was 8.40 (±2.07). At 12 years of age, 208 students were examined, the prevalence of caries found was 65.86%, the DMFT index was 1.85 (±2.22), the RI was 36.63%, the SiC index was 5.43 (±2.07), and children without the presence of periodontal pathology was 59.13%. At 15 years of age, 165 students were examined, the prevalence of caries was 70.06%, the DMFT was 3.08 (±3.39), the RI was 42.42%, the SiC index was 8.10 (±2.55), and adolescents without periodontal disease was 47.90%. Conclusions: Melilla-born children and adolescents had higher caries indicators and indices than the corresponding national averages for Spain. Teenagers under the age of 15 have a particularly high frequency of dental caries. The investigation of the children’s origin is where there is the most disparity. Children of Berber descent have much higher values than children of European heritage.

## 1. Introduction

Epidemiological studies are the main source of information on the frequency and distribution of conditions, health determinants and risk factors in populations. They provide us with the necessary data to carry out preventive and therapeutic programmes for the population studied [1].

In all the regions of Spain, with the exception of Melilla, there are epidemiological surveys on oral health. Their importance lies in the fact that they are the basis for decision-making by public health services. The measures adopted are aimed at achieving the objectives set by the Spanish Council of Dentists and Stomatologists for Spain for 2020 [2] and those set by the World Health Organization (WHO, Geneva, Switzerland) [3].

**Figure 1:**
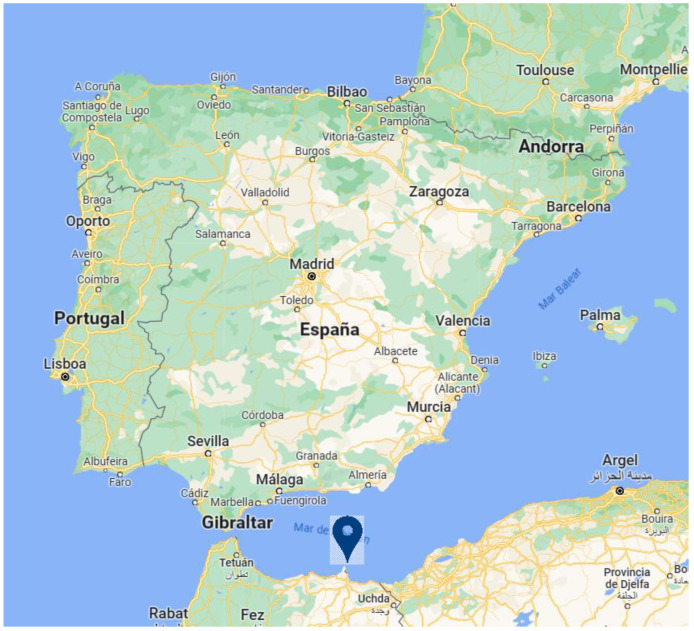
Geographical situation of Melilla (location taken from Google Maps).

The geographical importance of Melilla should be highlighted. This Spanish autonomous city of twelve square kilometres is located in the North of Africa. It can be seen on the map in Figure 1 [4]. It is bathed by the Mediterranean Sea and shares a border with Morocco. It is characterised by the multicultural nature of its population; Christians, Muslims, Jews and Hindus are part of its community. Due to this multiculturalism, there are large socioeconomic differences among its population that have a clear correspondence in health inequalities in general [5,6,7].

Because of Melilla’s proximity to Morocco, the Muslim population has grown in recent years and has reached almost 50% of the registered population [8]. Moreover, according to data from the latest census on 1 January 2019, the Moroccan resident population totals 13,259 out of 86,000 inhabitants, i.e., 15.3% [9]. In terms of the child population, it has the highest birth rate in Spain, and nearly 75% of births are in Muslim families [10].

In terms of oral and dental health care, the city’s only hospital provides a maxillofacial surgery service, and in 2020 a dental care service for special patients was incorporated.

Melilla has four health centres for primary care, but only one offers dental care. Two dentists who are members of the Melilla College of Dentists are in charge of this service, and two hygienists also work there [11]. 

The children’s oral health care programme is based on a public model regulated by Spanish law (RD 111/2008 of 1 January 2008) [12]. The therapeutic actions carried out in this Oral Care Unit are common to other similar programmes.

The main objective of this pioneering survey is to find out the main oral health problems in children and in this way propose a line of preventive and therapeutic action, which will make it possible to know if there are differences in oral health and hygiene habits between children and adolescents from different communities.

In this way, we will analyse the prevalence of caries, caries rates, restoration rate and community periodontal index (CPI) in cohorts established by WHO and establish a comparison with the targets set by the Council of Dentists for the year 2020 [2].

## 2. Materials and Methods

### 2.1. Study Design

A sampling plan was drawn up for carrying out an oral health survey in the Autonomous City of Melilla, taking as a reference the general recommendations established by the WHO regarding surveys in this area of health [13].

An oral health form was also developed based on the model proposed by WHO [14], with the aim of guaranteeing and standardising the results and to allow comparison of the data obtained in this study with other studies. This form was modified by eliminating the questionnaire on TMJ, attachment loss, prosthetic status and need for prostheses, and malocclusion data, and by adding the presence and severity of MIH in all cohorts.

In order to carry out the survey in schoolchildren, an appropriate authorisation was obtained from the Provincial Direction of Education and Science of the Autonomous City of Melilla, with verbal authorisation from the directors of the educational centres and with signed informed consent and authorisation from the parents or legal guardians.

### 2.2. Sample Selection

The reviews were carried out in schools in Melilla. For this purpose, we divided the city map geographically into 3 zones: north, centre and south. With a sample universe of about 22,000, a margin of error of 5% and a confidence level of 95%, the final sample size should be 378 children and adolescents. Nevertheless, finally, the sample was as follows, as shown in Table 1.

A total of 651 children were examined, divided into the following cohorts: 278 children belonged to the cohort of 6-year-olds (3rd year of infant school), 208 belonged to the 12-year-old cohort (6th year of primary school), and 165 belonged to the 15-year-old cohort (3rd year of secondary school).

(a) Inclusion criteria: All students from schools in Melilla included in the cohorts were included in the study.
(b) Exclusion criteria: Children were not included in the study if they had any type of physical or mental disability or oral malformation such as cleft lip.

### 2.3. Data Collection

Data collection was carried out by a single person. Intraexaminer agreement was established with a Kappa index of 0.90 according to the criteria of Landis and Koch [15].

It was carried out between October 2019 and March 2020 in the classrooms of the centres themselves.

The materials used were ambient light and direct light by means of an LED flashlight, a mirror LED torch, disposable intraoral flat mirror no. 5, WHO periodontal probe, gauze for drying the field, disposable latex gloves and masks.

### 2.4. Sociodemographic Variables of the Study

Several sociodemographic variables were collected in the overall study, such as gender, geographical area where the school or institute was located and socioeconomic level of the families.

In this article, we focus on the variable that may be of most interest: the origin of the children. For this purpose, we recorded whether the child was of Berber or European origin. This criterion was established on the basis of the child’s first name and surname.

The codes used were as follows:

1: European origin
2: Berber origin
3: Mixed origin of both cultures

### 2.5. Oral Health Variables

- Dental pathologies: presence or absence of caries (according to WHO 4th edition criteria), absence of teeth and presence of dental pathology (according to WHO 4th edition [16] criteria), absence of teeth and presence of molar incisor hypomineralisation (MIH pathology) (according to the criteria of the European Academy of Paediatric Dentistry in the 12-year-old cohort, classifying it into the following categories [17]).
- Need for treatment (according to WHO 4th edition criteria [16]).
- Periodontal pathologies: presence of bleeding on probing and presence or absence of calculus (according to WHO 4th edition criteria [16]).

### 2.6. Statistical Analysis

The database was cleaned to check the completeness and consistency of the information. Continuous variables are presented as means, medians and standard deviations. Categorical variables are presented as proportions and percentages. In order to identify the association between the presence of caries index and the independent variables, non-parametric Wilcoxon/Kruskal–Wallis tests (rank sums) were performed for continuous variables, and in the case of more than two categories, non-parametric tests were used for each pair (Wilcoxon method of non-parametric comparisons). Analysis of the chi-squared test was used to identify associations. In the case that the expected value of any of the cells in the continence table was less than five, Fisher’s chi-squared test for tetrachoric tables was applied. A statistically significant difference was considered to exist for *p* < 0.05. The STATA V15 package was used (STATA statistical software, College Station, TX, USA) for data analysis.

## 3. Results

### 3.1. Sample Distribution

Table 2 shows the distribution of the population studied according to the sociodemographic variables of sex, social status and origin, by age.

By origin, in the three ages studied, around 60% of the population was of Berber origin while approximately 30% was of European origin.

### 3.2. Caries Indices

The results obtained for the overall caries rates in the three cohorts studied are shown in Table 3. In this table, we show the mean number of decayed, missing and filled teeth for temporary and permanent dentition at the age of 5 to 6 years and for permanent only (DMFT) for 12 and 15 years. Similarly, the restorative index (percentage of filled teeth) and the significant caries index (SiC index) are shown, and this corresponds to the third cohort with the highest DMFT score.

In all cohorts, the component of the dft/DMFT index with the highest presence is the carious component. The absent component is almost nil at all three ages, and the obturated component is most prevalent at 15 years of age.

At 15 years of age, we found the highest DMFT index of the three cohorts with a value of 3.08 (±3.39).

The restorative index does not reach 50% in any of the cohorts, and for the 5–6-year-old group it is only 4.62% with an SD of ±16.94.

#### 3.2.1. Results by Origin

As we have seen in Table 2, of the total sample of 6-year-old children, 76 are of European origin (27.44%), 184 are of Berber origin (66.42%), and 17 are of mixed marriage origin (6.14%).

The 12-year-old children screened were distributed as follows: 76 were of European origin (36.54%), 116 were of Berber origin (55.77%), and 16 were of mixed marriage origin (7.69%).

The 15-year-old study group is distributed as follows: 76 adolescents were of European origin (40.12%), 116 were of Berber origin (55.69%), and 16 were of mixed marriage origin (4.19%). Table 4 shows the results obtained.

#### 3.2.2. Prevalence of Caries

We can verify that, at all ages, the children who presented the highest caries experience are the group of children whose origin is Berber. These children had approximately twice the caries prevalence of children of European origin, mainly at the age of 6 years in the primary dentition, where children of Berber origin had a prevalence of 65.95%, compared with 36.84% for children of European origin.

#### 3.2.3. dft/DMFT Index

Children of Berber origin present at all ages a higher dft/DMFT index than that found in children of European origin, with the greatest differences found at 6 and 15 years of age, becoming in the last cohort four times higher (DMFT of schoolchildren of Berber origin = 4.55 (±3.70) and the average DMFT of schoolchildren of European origin = 1.22 (±1.60)).

Within this index, the carious component is the one that represents the greatest weight, being, once again, greater in children of Berber origin. This difference is notable in the 6-year-old cohort in the primary dentition and in the 15-year-old cohort, where it is almost three times greater in children of Berber origin.

Of the three components of the index, the absent component is the one that represents the least volume at all ages. No statistically significant differences were found in any of them.

In the filled component, statistically significant differences were only found in the primary dentition at 6 years of age and in the permanent dentition at 15 years of age. In the latter group, children of Berber origin have a higher rate of fillings than adolescents of European origin.

#### 3.2.4. Restorative Index (RI)

At 6 years of age in the primary dentition, children from families of European origin present an average number of restorations of 18.80 (±33.76), while children of Berber origin only have an average number of restorations of 1.55 (±7.33).

At age 12, the children with the highest rate are children of European origin with an average of 56.18 (±48.63). This is followed by children from mixed marriages with an average value of 50.00 (±50.00), and, finally, the children with the lowest value are children of Berber origin with an average of 25.46 (±39.44).

### 3.3. Periodontal Index (CPI)

Table 5 shows the results for periodontal status.

At 12 years of age, we found that 59.13% of the children had a healthy periodontium, 25.48% of the children had haemorrhage or bleeding, and 15.38% of the children had calculus.

We found no significant differences in the CPI according to the origin of the children and adolescents between the three groups studied.

## 4. Discussion

To begin with, we would like to comment that this study has a series of limitations to be considered for the interpretation of the results obtained. These are inherent to the type of study carried out and are minimised by the standardisation of the criteria as set out by the WHO in its recommendations [16].

### 4.1. dft, DMFT Index and Caries Prevalence

In terms of caries prevalence, the results obtained show that in all cohorts, children of Berber origin have higher values. It should be noted that at 6 and 15 years of age, children of Berber origin have twice the caries prevalence of children of European origin. In none of the cohorts and none of the ethnic groups are the targets set by the General Council of Dentists for the year 2020 [2] met.

The dft/DMFT index is higher in children of Berber origin in all cohorts. At the age of 6 years, there is the least difference in the average dft/DMFT index between children of different ethnicities. The difference becomes more noticeable as the age of the children increases, reaching four times higher in the Berber population at 15 years of age.

Within the DMFT index, the component that stands out the most is the carious component, since in all cohorts it is the one with the greatest weight, being higher, again, in children and adolescents of Berber origin.

In the 2020 [18] national survey, only less than 2.1% of children in the 5–6-year-old, 12-year-old and 15-year-old cohorts were from African countries. This percentage is very low and unrepresentative of populations that could be similar to that of Melilla, making it impossible for us to compare the data from our survey with the children included at the national level.

In Melilla in 2016, Hadi [19] published a study on oral health in Melilla’s 6-year-old children in which special emphasis was placed on the oral health of the Muslim population of Melilla. This author obtained similar results to our study, where this population group had a higher prevalence of caries (52.65%) than children of European origin (28.39%).

In 2016, Gonçalves [20] conducted a study on the oral health of children in the Melilla Temporary Stay Centre. Most of the children were of Syrian origin, but children from other countries, including Morocco, were also examined. The average DMFT index for the ages studied (5 to 13 years) was 3.3. This average is not far from the result obtained in our study for children of Berber origin, where at the age of 6 years the average DMFT index is 3.61, at the age of 12 years it is 2.40, and at the age of 15 years it is 4.55.

The 1996 Ceuta survey [21] collected data on Muslim children (comparable to those of Berber ethnicity in our survey) and on non-Muslim children (comparable to those of European origin in our survey). The results obtained are that in all cohorts there are statistically significant differences between Muslim and non-Muslim children, with the former presenting much higher values for the DMFT index.

At 6 years of age in Ceuta, the percentage of children with DMFT > 1 was 84% in Muslim children compared with 50.7% in non-Muslim children. At this age, ethnicity statistically influenced the cod index (4.40 for Muslim children and 2.07 for non-Muslim children).

By age 12, the percentage of children with DMFT > 1 was also higher in Muslim children (93.2%) than in non-Muslim children (79.7%). The DMFT index followed the same trend as in the previous cohort with statistically significant differences: 5.00 in Muslim children versus 3.26 in non-Muslim children.

By the age of 15, the percentages in Ceuta are closer between the two groups, but the percentage of children with DMFT > 1 is still higher in Muslims than in non-Muslims (86.3% and 72.5%, respectively). At this age, there is no statistically significant difference between the two groups in the DMFT index (5.30 in Muslim children and 4.20 in non-Muslim children).

As it can be seen, the results of our study are similar to those found in Ceuta: In all cohorts, caries rates and caries prevalence are higher in children of Berber origin (Table 6).

The Berber ethnic group extends throughout North Africa, from the Canary Islands to Egypt [22,23]. Hence, the children of this ethnic group screened in our city present similar characteristics in terms of customs and healthy habits to the countries of this geographical area.

The closest Berber country to Melilla is Morocco, and many of the children included in our study have a deep-rooted culture from this neighbouring country [24]. It is therefore interesting to note that for these children, the results we have obtained are better and have improved on those found in the National Survey carried out by the Moroccan Ministry of Health in 2012 [25].

In Morocco, at 12 years of age, the DMFT was 4.82 and the prevalence of caries was 81.8%, while in the study in Melilla we obtained results of DMFT of 2.40 and caries prevalence of 73.8%.

For the 15-year-olds, the results are more similar: In Morocco, the DMFT index was 5.54 with a prevalence of 86.67%, and in our study the result for this cohort was 4.55 with a prevalence of 86.67%.

We can also compare the results for the Berber population studied in Melilla with results obtained in the second closest country to our city, Algeria [26], in 2013. In this country, the DMFT index was 2.55 for 12-year-olds and 3.45 for the 15-year-old cohort.

Table 7 compares the results of the surveys in Morocco and Algeria with the results found in our survey of ethnic Berber children and adolescents in Melilla.

### 4.2. Restorative Index

As we have seen above, the RI serves as an indicator of the quality of oral health services offered to the general population [27]. In our case, the study shows that RI for children of European origin in all cohorts reaches acceptable levels, while for children of Berber ethnicity the results are much worse.

This may be due to the fact that, in Melilla, children of Berber origin, as a general rule and with some exceptions, are associated with lower socioeconomic levels and reside in the most vulnerable areas [28], making access to private clinics to treat pathology more complicated [29,30], which demonstrates the deficit of therapeutic care provided by the public health system.

The study conducted in 2016 on the oral health of Melilla’s 6-year-old children [19] does not provide us with any data on the rate of oral health care provided by the public health system, so we cannot make any comparisons with data prior to our study.

In the study by Gonçalves [20] on children at the CETI in Melilla, the overall RI (mean for all ages studied) was 5.6 for the group including Moroccan children. In our study, the index for children of Berber origin is higher, reaching a value of 37.11 for the 15-year-old cohort.

This fact indicates that adolescents of Berber origin in Melilla have easier access to dental treatment than the children at the CETI.

In Ceuta, in 1996 [21], no relationship was established between the RI of the children according to whether they were Muslim or non-Muslim. Only the RI’s association with the variable of active or non-active parents is referred to. So, again, we cannot make a comparison between the children in our sister city and the children in Melilla.

The studies carried out in Moroccan cities, likewise, do not reveal any data on the restoration rate of the child population, but only a study of the prevalence and rates of caries.

### 4.3. Periodontal Index (CPI)

In the CPI study for the 12- and 15-year-old cohorts, we observed no statistically significant differences between the two groups of children by origin. In both cohorts, the Oral Health Objectives set by the Council of Dentists for the year 2020 [2] are met.

In the study of the oral health of children in Ceuta in 1996 [21], the CPI for Muslim children (for all ages) was 52% healthy, 22% with bleeding on probing and 26% with calculus. For non-Muslim children, the percentage of healthy children was 62.50%, children with bleeding on probing was 15.60%, and for children with calculus the percentage was 21.90%. These differences between the two groups were not statistically significant.

These results, on the whole, are similar to ours, as none of them show statistically significant differences, and non-Muslim children (those of European origin in our study) show better results than Muslim children (of Berber ethnicity in our study).

In Morocco, the study by Zaoui [31] obtained results for the periodontal index in children aged 12 years: 45% of children in good periodontal health, 20% of children with bleeding on probing and 30% with calculus.

In our study for the same age group, the results are similar in children of Berber origin. The only component that differs is bleeding on probing, as in our study, unlike that of Zaoui [31], it is less than children with calculus.

The 2012 national survey in Morocco [25] established that 42.3% of children aged 12 years had some degree of periodontal pathology, and in adolescents aged 15 years this percentage was 59.7%. In our study, we obtained the same results in the Berber population (42.24% of 12-year-olds and 58.91% of 15-year-olds had periodontal pathology) (Table 8).

## 5. Conclusions

This epidemiological study shows an improved oral health status in terms of caries and periodontal disease in the infant–juvenile population of Melilla, which should be taken into account when developing oral health programmes for the affected population.

The prevalence and rates of caries are considerably higher in children of Berber origin, who have a higher prevalence and rates of caries than children of European origin. Only 12-year-old children of both ethnicities and 15-year-old children of European origin meet the objectives related to periodontal health set by the Spanish authorities.

## Figures and Tables

**Table 1:** Final sample, divided by areas and age.

	6 Years Old	12 Years Old	15 Years Old	Total
North area	116	59	27	202
Centre area	93	71	57	221
South area	69	78	81	228
Total	278	208	165	651

**Table 2:** Sample distribution.

	5–6 Years		12 Years		15 Years	
	*n*	(%)	*n*	(%)	*n*	(%)
Total	278	(100)	208	(100)	165	(100)
Sex						
Boys	119	(42.80)	98	(47.11)	67	(40.60)
Girls	159	(57.19)	110	(52.80)	98	(59.30)
Social Level						
High-medium	138	(49.64)	149	(71.63)	137	(83.03)
Low	140	(50.36)	53	(28.37)	28	(16.97)
Origin						
European	76	(27.34)	76	(36.54)	67	(40.60)
Berber	185	(66.55)	116	(55.77)	91	(55.15)
Mixed	17	(6.12)	16	(7.69)	7	(4.24)
Geographical area						
Centre area	93	(33.45)	71	(34.13)	57	(34.55)
North area	116	(41.73)	59	(28.37)	27	(16.36)
South area	69	(24.82)	27	(37.50)	81	(49.09)

**Table 3:** Caries indices divided by age.

					RI (%)		dft/DMFT		SiC Index	
	*n*	Decayed	Missing	Filled	RI	±SD	Mean	±SD	Mean	±SD
5–6 years temporary	278	2.57		0.12	4.62	±16.94	2.77	±3.44	8.40	±2.07
5–6 years permanent	278	0.17		0.01			0.19	±0.63		
12 years	208	1.39	0.01	0.47	36.63	±45.25	1.85	±2.22	5.43	±2.07
15 years	165	1.86	0.10	1.12	42.42	±41.88	3.08	±3.39	8.10	±2.55

**Table 4:** Results by origin and age.

	Origin	6 Years Temporary		6 Years Permanent		12 Years		15 Years	
Caries Prevalence	European	36.84%		17.86%		53.95%		58.21%	
	Berber	65.95%		78.57%		73.28%		81.72%	
	Mixed	29.41%		5.88%		68.75%		28.57%	
	*p*-value	<0.0001		0.3367		0.0223		0.0003	
		Mean	±SD	Mean	±SD	Mean	±SD	Mean	±SD
DMFT/dft Index	European	1.11	±2.03	0.11	±0.42	1.12	±0.42	1.22	±1.60
	Berber	3.61	±3.71	0.23	±0.71	2.40	±0.71	4.55	±3.70
	Mixed	0.94	±1.64	0.12	±0.49	1.69	±0.49	0.86	±1.57
	*p*-value	<0.0001		0.3594		0.0003		<0.0001	
Decayed	European	0.83	±1.59	0.08	±0.36	0.66	±1.52	0.66	±1.33
	Berber	3.42	±3.50	0.22	±0.68	1.92	±2.36	2.76	±2.88
	Mixed	0.94	±1.64	0.11	±0.49	1.00	±1.93	0.43	±1.13
	*p*-value	<0.0001		<0.0001		<0.0001		<0.0001	
Missing	European	0.03	±0.16			0.03	±0.16	0.01	±0.12
	Berber	0.10	±0.44			0.01	±0.09	0.15	±0.59
	Mixed	0.00	±0.00			0.00	±0.00	0.14	±0.38
Filled	European	0.25	±0.83	0.22	±0.03	0.43	±0.72	0.55	±0.94
	Berber	0.08	±0.51	0.10	±0.01	0.47	±0.92	1.64	±2.32
	Mixed	0.00	±0.00	0.00	±0.00	0.69	±1.49	0.29	±0.76
	*p*-value	0.0395						0.0055	
RI (Restorative Index)	European	18.80	±33.76			56.18	±48.63	52.38	±46.15
	Berber	1.55	±7.33			25.46	±39.44	37.11	±38.52
	Mixed	0.00	±0.00			50.00	±50.00	50.00	±70.71
	*p*-value	0.0002				0.0037			

**Table 5:** Results by CPI, age and origin.

	Origin	12 Years		15 Years	
		*n*	%	*n*	%
Healthy	European	48	63.16	35	52.24
	Berber	67	57.76	41	44.09
	Mixed	8	50.00	4	57.14
	TOTAL	123	59.13	80	47.9
Bleeding	European	14	18.42	20	29.85
	Berber	35	30.17	29	31.18
	Mixed	4	25.00	1	14.29
	TOTAL	53	25.48	50	29.94
Calculus	European	14	18.42	12	17.91
	Berber	14	12.07	23	24.73
	Mixed	4	25.00	2	28.57
	TOTAL	32	15.38	37	22.16

**Table 6:** Comparison of dft/DMFT index by origin or ethnicity between Melilla and Ceuta.

	Year	Age	dft/DMFT Index		Prevalence (%)	
			European	Berber	European	Berber
Melilla	2020	6 years temp	1.11	3.61	36.84	65.95
Melilla: Hadi [19]	2016	6 years temp			28.39	52.65
Ceuta [22]	1996	6 years temp	2.07	4.40	50.7	84
Melilla	2020	12 years	1.12	2.40	53.95	73.28
Ceuta [21]	1996	12 years	3.23	5.00	79.7	93.2
Melilla	2020	15 years	1.22	4.55	58.21	81.72
Ceuta [21]	1996	14 years	4.20	5.30	72.5	86.3

**Table 7:** Comparison between the results of the dft/DMFT index and caries prevalence from the Moroccan and Algerian surveys and the results found in Melilla.

Country	Author	Year	Age	Caries Prevalence (%)	dft/DMFT
Spain (Melilla)		2020	6	65.95	3.61
Algeria	Public health institute	2013	6	77.10	3.5
Spain (Melilla)		2020	12 years	73.28	2.40
Morocco	Ministry of health	2012	12 years	81.80	4.82
Algeria	Public health institute	2013	12 years	72.20	2.55
Spain (Melilla)		2020	15 years	81.72	4.55
Morocco	Ministry of health	2012	15 years	86.67	5.54
Algeria	Public health institute	2013	15 years	73.10	3.45

**Table 8:** CPI scores in Melilla’s ethnic Berber children, Muslim children from Ceuta and African countries at 12 and 15 years of age.

Country	Year	Age	Healthy (%)	Bleeding (%)	Calculus (%)
Melilla	2020	12	57.76	30.17	12.07
Morocco [31]		12	45	20	30
National survey Morocco	2012	12		62.5	
Ceuta	1996	12–14	52	22	26
Melilla	2020	15	44.09	31.18	27.73
National survey Morocco	2012	15		71.2	

## Data Availability

Data will be available by asking the corresponding authors.

## References

[1] Celentano D.D., Szklo M. (2018). Gordis Epidemiology.

[2] Llodra Calvo J.C., Bourgeois D. (2009). Estudio Prospectivo Delphi la Salud Bucodental en España 2020. Tendencias y Objetivos de Salud Oral.

[3] Hobdell M., Petersen P.E., Clarkson J., Johnson N. (2003). Global goals for oral health 2020. Int. Dent. J..

[4] Ciudad Autónoma de Melilla, Consejería de Fomento Plan General Documento Inicio Evaluación Ambiental Estratégica. https://www.melilla.es/melillaportal/RecursosWeb/DOCUMENTOS/1/0_24665_1.pdf.

[5] Mohatar Marzok M. (2009). Marroquíes en Andalucía: Dinámicas Migratorias y Condiciones de Vida.

[6] Cámara Gorgé R. Cultura, Religión y Sociedad. https://www.unirioja.es/.

[7] Consejería de Infraestructuras, Urbanismo y Depotes. https://carreraafricana.com/melilla-tierra-deculturas/.

[8] Observatorio Andalusi Estudio Demográfico de la Población Musulmana. Exploaticón Estadistica de Censo de Ciudadanos Musulmanes en España referido a Fecha de 31/12/2019. http://observatorio.hispanomuslim.es/estademograf.pdf.

[9] Instituto Nacional de Estadística (2019). Avance de la Estadística del Padrón Continuo a 1 de Enero de 2019 Datos Provisionales.

[10] (2016). El 75 Por Ciento de los Nacimientos en Melilla ya Son Musulmanes: El Sábado Nacieron 15 Marroquíes y un Solo Español. http://www.alertadigital.com/2016/12/05/el-75-por-ciento-de-los-nacimientosen-melilla-ya-sonmusulmanes-el-sabado-nacieron-15-marroquies-y-un-solo-espanol/.

[11] Área de Salud Melilla Instituto Nacional de Gestión Sanitaria (2020). Unidades de Apyo/Unidad de Salud Bucodental. Área salud Melilla. http://www.areasaludmelilla.es/asm/index.php.

[12] Real Decreto 111/2008, de 1 de Febrero, Por el Que se Regula la Concesión Directa de Subvenciones a Las Comunidades Autónomas Para la Promoción de Actividades Para la Salud Bucodental Infantil Durante el Año 2008 «BOE» núm. 31, de 5 de Febrero de 2008, Páginas 6265 a 6267 (3 págs.) Sección: I. Disposiciones Generales Departamento: Ministerio de Sanidad y Consumo Referencia: BOE-A-2008-1904. https://www.boe.es/eli/es/rd/2008/02/01/111.

[13] OMS (2013). Encuestas de Salud Bucal: Métodos Básicos.

[14] Rubio J.M., Salazar F.S., Osés J.A., González V.L. (1997). Criterios Mínimos para los Estudios Epidemiológicos de la Salud Dental en Escolares. Rev. Española Salud Pública.

[15] Landis J.R., Koch G.G. (1977). The measurement of observer agreement for categorical data. Biometrics.

[16] WHO (1997). Oral Health Surveys: Basic Methods.

[17] Weerheijm K.L., Duggal M., Mejàre I., Papagiannoulis L., Koch G., Martens L.C., Hallonsten A.L. (2003). Judgement criteria for molar incisor hypomineralisation (MIH) in epidemiologic studies: A summary of the European meeting on MIH held in Athens, 2003. Eur. J. Paediatr. Dent..

[18] Bravo Pérez M., Almerich Silla J.M., Canorea Díaz E., Casals Peidró E., Cortés Martinicorena F.J., Expósito Delgado A.J., Gómez Santos G., Hidalgo Olivares G., Lamas Oliveira M., Martínez Beneyto Y. (2020). Encuesta nacional de salud oral en España 2020. Rev. Española Odontol..

[19] El Hadi Al-Lal M., Estévanez Botello M.A., Casas Casas A., León Hurtado M.A. (2019). Estudio de Salud Bucodental Infantil en la Ciudad Autónoma de Melilla. Rev. Higienistas.com.

[20] Gonçalves Riatto S., Montero J., Ribas Pérez D., Castaño-Séiquer A., Dib A. (2018). Oral Health Status of Syrian Children in the Refugee Center of Melilla, Spain. Int. J. Dent..

[21] Nieto García V.M., Nieto García M.A., Lacalle Remigio J.R., Abdel-Kader Martín L. (2001). Salud oral de los escolares de ceuta. influencias de la edad, el género, la etnia y el nivel socioeconómico. Rev. Española Salud Pública.

[22] Algora Weber M.D. (2015). Minorías y Fronteras en el Mediterráneo Ampliado: Un Desafío a la Seguridad Internacional del Siglo XXI.

[23] Galand L. (2004). Les Berbères Dans L’histoire: Mélanges Offerts à Gabriel Camps.

[24] Marmolejo J.A., Plata Díaz A.M. (2018). Análisis Cultural de la Ciudad Autónoma de Melilla Factores y Consecuencias Para su Desarrollo Social y Económico.

[25] (2018). Un Plan Stratégique National de Développement du Secteur de la Sante Buccodentaire Pour la Période 2018–2025. https://maroc-diplomatique.net/un-plan-strategique-national-de-developpement-dusecteur-de-la-sante-buccodentaire/.

[26] Institut National de Santé Publique (2013). Etat de Santé Bucco-Dentaire de L’enfant Algérien 6 Ans, 12 Ans et 15 Ans.

[27] Frencken J.E., Peters M.C., Manton D.J., Leal S.C., Gordan V.V., Eden E. (2012). Minimal intervention dentistry for managing dental caries—A review: Report of a FDI task group. Int. Dent. J..

[28] PROMESA, Ciudad Autónoma de Melilla Diagnóstico del Área de Estructura Social. https://www.planestrategicomelilla.net/f12_02.htm.

[29] Petersen P.E., Kandelman D. (2013). Equitable oral health for all: The role of oral health promotion. Bull. World Health Organ..

[30] Sabbah W., Tsakos G., Chandola T., Sheiham A., Watt R.G. (2007). Social gradients in oral and general health. J. Dent. Res..

[31] Zaoui F., Hamdani S., Belhad M.J., Miquel J.L. (1996). Etude descriptive de l’état bucco-dentaire d’un échantillon de la population marocaine. Rev. Odont. Stomatol. Trop..

